# 

**Published:** 1866-04

**Authors:** 


*Journals Received.*—Savannah *Journal of Medicine*—new series, edited by Drs. Juriah Harris, James B. Read and J. G. Thomas, a monthly of 68 pages. Published in Savannah, Georgia.

*Richmond Medical Journal*, a monthly of 78 pages, edited by E. S. Gailliard, M. D., and W. S. McChesney, M. D., Richmond, Virginia. The Boston *Medical and Surgical Journal*, a weekly of 20 pages, edited by Samuel L. Abbot, M. D., and James C. White, M. D., Boston, Massachusetts. The New York *Medical Journal*, a monthly record of medicine and the collateral sciences. The Chicago *Medical Examiner*, a monthly of 64 pages, edited by N. S. Davis, M. D., Chicago, Ill. The Cincinnati *Lancet and Observer*, a monthly of 68 pages, edited by Edward B. Stevens, M. D., and John A. Murphy, M. D., Cincinnati, Ohio. Buffalo *Medical and Surgical Journal*, a monthly of 56 pages, edited by Julius F. Miner, M. D., Buffalo, N. Y. The Detroit *Review of Medicine and Pharmacy*, editors, George P. Anderson, M. D., Samuel P. Duffield, Ph. D., and Edward W. Jenks, M. D., Detroit, Mich. The *Medical and Surgical Reporter*, a weekly journal of 26 pages, S. W. Butler, M. D., editor, Philadelphia. The Cincinnati *Journal of Medicine*, Geo. C. Blackman, M. D., T. Parvin, M. D., and R. Bartholow, M. D., editors. The *Medical and Surgical Monthly*, Memphis, Tennessee, Frank Ramsey, M. D. editor.

*List of Payments*.—Drs. G. T. Pursley, $5; F. M. Brantly, $4; John T. Dixon, $4 36; J. J. Knott, $2; Smith & Edwards, $4; Turner, $4; J. G. W. Brown, $4; N. F. Howard, $4; C. L. Redwine, $4; D. H. Conally, $4; Taylor & Ball, $4; G. G. Crawford, $4; J. M. Johnson, $4; J. L. Westbrook, $4; G. B. Douglas, $4; D. C. O'Keefe, $4; W. P. Harden, $4; E. J. Roach, $4; T. S. Powell, $4; Alexander & Orme, $4; W. B. Wells, $4; J. P. Hervey, $4; J. J. Cooper, $4; Wm. H. Pilcher, $4; F. M. Thomason, $2; Connally & Cromell, $4; Taliaferro, $4; Davis & Fitts, $4.

